# Predicting hospital stay, mortality and readmission in people admitted for hypoglycaemia: prognostic models derivation and validation

**DOI:** 10.1007/s00125-017-4235-1

**Published:** 2017-03-17

**Authors:** Francesco Zaccardi, David R. Webb, Melanie J. Davies, Nafeesa N. Dhalwani, Laura J. Gray, Sudesna Chatterjee, Gemma Housley, Dominick Shaw, James W. Hatton, Kamlesh Khunti

**Affiliations:** 1 Diabetes Research Centre, University of Leicester, Leicester General Hospital, Gwendolen Road, Leicester, LE5 4PW UK; 2 Department of Health Sciences, University of Leicester, University Road, Leicester, UK; 3 Nottingham University Hospitals & East Midlands Academic Health Science Network, Triumph Road, Nottingham, UK; 4 Nottingham Respiratory Research Unit, University of Nottingham, Hucknall Road, Nottingham, UK

**Keywords:** Epidemiology, Hypoglycaemia, Inpatient, Mortality, Prognostic model

## Abstract

**Aims/hypothesis:**

Hospital admissions for hypoglycaemia represent a significant burden on individuals with diabetes and have a substantial economic impact on healthcare systems. To date, no prognostic models have been developed to predict outcomes following admission for hypoglycaemia. We aimed to develop and validate prediction models to estimate risk of inpatient death, 24 h discharge and one month readmission in people admitted to hospital for hypoglycaemia.

**Methods:**

We used the Hospital Episode Statistics database, which includes data on all hospital admission to National Health Service hospital trusts in England, to extract admissions for hypoglycaemia between 2010 and 2014. We developed, internally and temporally validated, and compared two prognostic risk models for each outcome. The first model included age, sex, ethnicity, region, social deprivation and Charlson score (‘base’ model). In the second model, we added to the ‘base’ model the 20 most common medical conditions and applied a stepwise backward selection of variables (‘disease’ model). We used C-index and calibration plots to assess model performance and developed a calculator to estimate probabilities of outcomes according to individual characteristics.

**Results:**

In derivation samples, 296 out of 11,136 admissions resulted in inpatient death, 1789/33,825 in one month readmission and 8396/33,803 in 24 h discharge. Corresponding values for validation samples were: 296/10,976, 1207/22,112 and 5363/22,107. The two models had similar discrimination. In derivation samples, C-indices for the base and disease models, respectively, were: 0.77 (95% CI 0.75, 0.80) and 0.78 (0.75, 0.80) for death, 0.57 (0.56, 0.59) and 0.57 (0.56, 0.58) for one month readmission, and 0.68 (0.67, 0.69) and 0.69 (0.68, 0.69) for 24 h discharge. Corresponding values in validation samples were: 0.74 (0.71, 0.76) and 0.74 (0.72, 0.77), 0.55 (0.54, 0.57) and 0.55 (0.53, 0.56), and 0.66 (0.65, 0.67) and 0.67 (0.66, 0.68). In both derivation and validation samples, calibration plots showed good agreement for the three outcomes. We developed a calculator of probabilities for inpatient death and 24 h discharge given the low performance of one month readmission models.

**Conclusions/interpretation:**

This simple and pragmatic tool to predict in-hospital death and 24 h discharge has the potential to reduce mortality and improve discharge in people admitted for hypoglycaemia.

**Electronic supplementary material:**

The online version of this article (doi:10.1007/s00125-017-4235-1) contains peer-reviewed but unedited supplementary material, which is available to authorised users.

## Introduction

Hypoglycaemia is the most common side effect of intensive glucose treatment [1]. Severe hypoglycaemic episodes have a negative impact on the quality of life of patients with diabetes and are possibly associated with an increased risk of vascular and nonvascular death [2, 3]. Most hypoglycaemic events are mild and self-treated; severe episodes, conversely, require third party support and some result in emergency department and hospital admission, which represents a significant burden on patients and healthcare systems [1, 4].

In recent years, several risk prediction models have been proposed for patients admitted to hospital; these models can assist clinicians to define the prognosis and tailor medical decisions [5]. The majority of models have been developed and validated for risk of inpatient death, length of hospital stay and hospital readmission in different clinical settings, mainly in patients with cardiovascular diseases [6–10]. To date, however, no model has been developed for patients admitted to hospital for hypoglycaemia. As these admissions are generally characterised by a lower risk of death (2–4%) and a shorter length of stay (usually <24 h) compared with admissions for other medical reasons [6, 11–14], the applicability of available prediction models to patients admitted for hypoglycaemia would result in biased risk estimates. Moreover, a risk prediction tool for patients admitted for hypoglycaemia would be particularly useful given the increased trend of patients admitted for hypoglycaemia during the last decade in England and the USA, and the associated use of healthcare resources [4, 11, 15].

In this context, we used Hospital Episode Statistics (HES) admission data from England to develop and validate a risk model for inpatient death, 24 h discharge and one month readmission for people admitted to hospital for hypoglycaemia.

## Methods

### Study design, setting and source of data

We extracted data from HES, which contains information on all finished consultant episodes in the National Health Service (NHS) hospital trusts in England (www.hscic.gov.uk/hes, accessed 29 November 2016). All hospital admissions reporting the ICD-10 (www.who.int/classifications/icd/en/) diagnosis field E160 (drug-induced hypoglycaemia without coma), E161 (other hypoglycaemia) or E162 (hypoglycaemia, unspecified) in the first position (i.e. hypoglycaemia as the primary reason of admission), and E10+ (insulin-dependent diabetes mellitus) or E11+ (non-insulin-dependent diabetes mellitus) in any of the remaining ICD-10 fields (from second to 20th), were included.

For each admission episode, we collected data on age, sex, self-reported ethnicity, region of usual residence, start and end date of the episode, admission and discharge method (reporting whether admission resulted in death) and Index of Multiple Deprivation (IMD, a weighted index of social deprivation). We used ICD-10 codes to calculate the Charlson comorbidity score [16]. For the current analysis, we defined two temporally distinct derivation and validation samples. In the derivation samples, we included admissions in 2013 for inpatient death and 2010–2012 for one month readmission and length of hospitalisation (defined as 24 h discharge). Corresponding years for validation samples were 2014 and 2013–2014. We selected these time intervals because trends of hospital admissions for hypoglycaemia were more stable during these years [11].

### Models specification

We modelled the three outcomes, inpatient death, one month readmission for hypoglycaemia and 24 h discharge, using complete-case logistic regressions. We developed two prognostic models: the first, defined ‘base’ model, included age (transformed with a cubic spline with five knots to account for the non-linearity of the relationship between age and hospital admission for hypoglycaemia [11]), sex, ethnicity (white, other), region (East Midlands, London, North East, North West, South East, South West, West Midlands, and Yorkshire and the Humber), social deprivation (deciles of IMD) and Charlson score for all three outcomes. In the second model (‘disease’), we added the 20 most common ICD-10 comorbidities reported in positions second to sixth to the base model; comorbidities were identified for each outcome and are reported in ESM Table 1. After their inclusion, we performed a stepwise backward elimination of individual factors using the ordinary Akaike’s information criterion to define the final set of variables [17].

### Model performance

We evaluated the performance of regression models assessing Nagelkerke *R* ^2^, discrimination and calibration. For a specific model, *R* ^2^ indicates the additional variation in the outcomes compared with a model with only the intercept. For a logistic regression, discrimination corresponds to the area under the receiver operating characteristic curve (C-index); a value of 0.5 indicates model discrimination no better than chance, while a value of 1 perfect discrimination [18]. We plotted observed outcomes by decile of predictions to graphically assess calibration, and calculated calibration slope and intercept; values around 1 for slope and 0 for intercept indicate correct calibration [19].

### Internal and temporal validation

We validated models internally with 300 bootstrap samples to assess possible optimism and temporally by recalculating indices of discrimination, plotting observed vs predicted outcomes, and estimating calibration slope and intercept. Finally, we developed a calculator based on recalibrated models using the calibration slope and intercept obtained in validation samples [20].

### Guidelines and software for analyses

We performed analyses following the general framework proposed by Steyerberg [20] and Harrell [21], and reported results in line with TRIPOD (Transparent Reporting of a multivariable prediction model for Individual Prognosis Or Diagnosis) recommendations (www.tripod-statement.org. accessed 29 November 2016). We used Stata 14.1 and R 3.2.3 (package rms [22]) for all analyses and reported results with 95% CI. A *p* value <0.05 was considered statistically significant.

## Results

### Characteristics of derivation and validation samples

Of 22,113 available admissions for inpatient death, one admission (0.005%) was excluded due to missing data on age. Of 55,978 available admissions for one month readmission and 24 h discharge, 41 admissions were excluded for one month readmission (0.073%; one missing information for age and 40 for social deprivation) and 68 for length of hospital stay (0.121%; one missing information for age, 34 for social deprivation, 27 for time to discharge and six for both social deprivation and time to discharge).

**Fig. 1:**
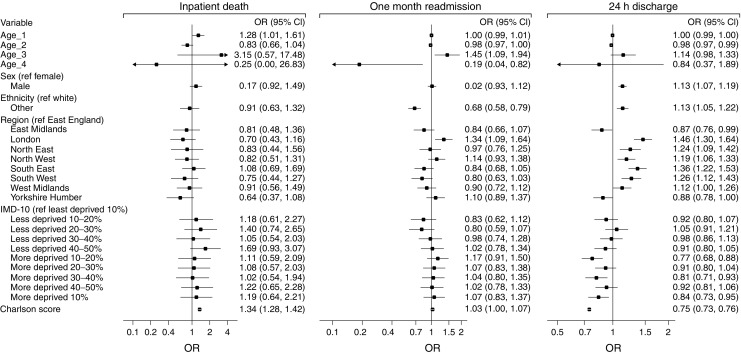
Associations of variables with outcomes for base model (derivation samples). Age_1, Age_2, Age_3 and Age_4 indicate restricted cubic spline transformation of age. ORs are reported per unit increase of Charlson score. Constants of the models were: -16.864 for inpatient mortality, -2.701 for one month readmission and 0.086 for 24 h discharge

**Fig. 2:**
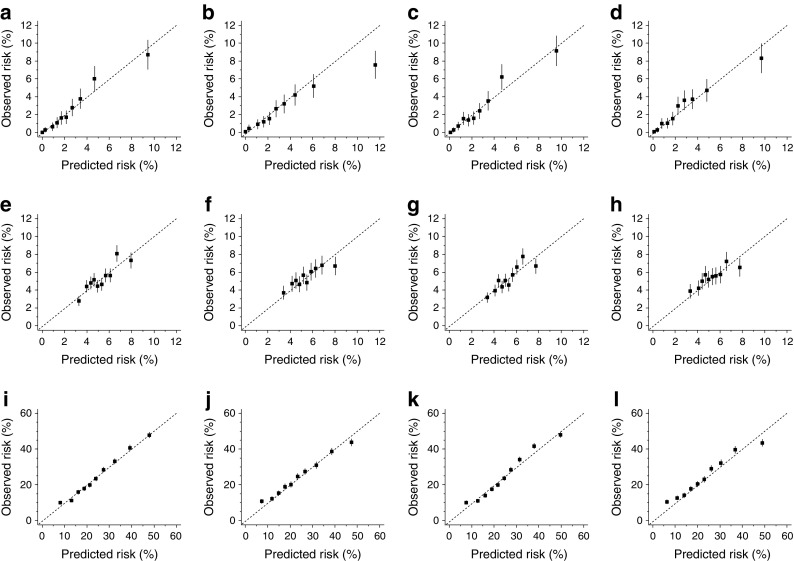
Calibration plots for base and disease models in derivation and validation samples. Inpatient death: base model, derivation (**a**) and validation (**b**) sample; disease model, derivation (**c**) and validation (**d**) sample. One month readmission: base model, derivation (**e**) and validation (**f**) sample; disease model, derivation (**g**) and validation (**h**) sample. 24 h discharge: base model, derivation (**i**) and validation (**j**) sample; disease model, derivation (**k**) and validation (**l**) sample. Error bars indicate 95% CI

Characteristics of the remaining admissions with complete data, by outcome and sample, are reported in Table 1. No major differences were found between the derivation and validation samples. A large proportion of admissions occurred in patients older than 60 years and of white ethnicity. There were slightly more admissions in men than women and Charlson scores were slightly higher in validation samples. The outcome-specific top 20 most common diseases covered approximately 50% of all reported comorbidities (ESM Table 1). Of these, two for readmission and 15 for 24 h discharge were included in the final models after the stepwise backward elimination. Associations between variables and outcomes in derivation samples are reported in Fig. 1 for base models and ESM Table 2 for disease models. Performance measures are summarised in Table 2 and calibration plots are depicted in Fig. 2.

**Table 1:** Characteristics of admissions to hospital for hypoglycaemia

Characteristic	Derivation			Validation		
	Inpatient death	One month readmission	24 h discharge	Inpatient death	One month readmission	24 h discharge
Calendar year	2013	2010–2012	2010–2012	2014	2013–2014	2013–2014
Admission, *n*	11,136	33,825	33,803	10,976	22,112	22,107
Participant, *n*	9937	28,554	28,533	9819	19,057	19,054
Death, *n*	296	–	–	296	–	–
Readmission, *n*	–	1789	–	–	1207	–
24 h discharge, *n*	–	–	8396	–	–	5363
Age at admission, years						
<20	638 (5.7)	2121 (6.3)	2121 (6.3)	594 (5.4)	1232 (5.6)	1232 (5.6)
20–29	334 (3.0)	1182 (3.5)	1182 (3.5)	344 (3.1)	678 (3.1)	678 (3.1)
30–39	395 (3.6)	1207 (3.6)	1206 (3.6)	341 (3.1)	736 (3.3)	736 (3.3)
40–49	678 (6.1)	2008 (5.9)	2007 (5.9)	686 (6.3)	1364 (6.2)	1362 (6.2)
50–59	964 (8.7)	2730 (8.1)	2730 (8.1)	960 (8.8)	1924 (8.7)	1924 (8.7)
60–69	1542 (13.9)	4348 (12.9)	4347 (12.9)	1468 (13.4)	3010 (13.6)	3010 (13.6)
70–79	2818 (25.3)	9120 (27.0)	9118 (27.0)	2840 (25.9)	5658 (25.6)	5656 (25.6)
≥80	3767 (33.8)	11,109 (32.8)	11,092 (32.8)	3743 (34.1)	7510 (34.0)	7509 (34.0)
Sex						
Women	5430 (48.8)	16,518 (48.8)	16,506 (48.8)	5257 (47.9)	10,687 (48.3)	10,686 (48.3)
Men	5706 (51.2)	17,307 (51.2)	17,297 (51.2)	5719 (52.1)	11,425 (51.7)	11,421 (51.7)
Charlson index	2.29 ± 1.62	2.04 ± 1.46	2.04 ± 1.46	2.37 ± 1.61	2.33 ± 1.62	2.33 ± 1.62
IMD-10^a^						
Least deprived 10%	607 (5.5)	1828 (5.4)	1826 (5.4)	633 (5.8)	1240 (5.6)	1240 (5.6)
Less deprived 10–20%	768 (6.9)	2241 (6.6)	2240 (6.6)	671 (6.1)	1439 (6.5)	1439 (6.5)
Less deprived 20–30%	857 (7.7)	2459 (7.3)	2458 (7.3)	806 (7.3)	1663 (7.5)	1663 (7.5)
Less deprived 30–40%	917 (8.2)	2863 (8.5)	2861 (8.5)	842 (7.7)	1759 (8.0)	1759 (8.0)
Less deprived 40–50%	1027 (9.2)	3108 (9.2)	3103 (9.2)	949 (8.7)	1976 (8.9)	1976 (8.9)
More deprived 10–20%	1436 (12.9)	4747 (14.0)	4743 (14.0)	1563 (14.2)	2999 (13.6)	2999 (13.6)
More deprived 20–30%	1359 (12.2)	4223 (12.5)	4222 (12.5)	1369 (12.5)	2728 (12.3)	2727 (12.3)
More deprived 30–40%	1195 (10.7)	3538 (10.5)	3537 (10.5)	1192 (10.9)	2387 (10.8)	2387 (10.8)
More deprived 40–50%	1101 (9.9)	3314 (9.8)	3312 (9.8)	1174 (10.7)	2275 (10.3)	2273 (10.3)
Most deprived 10%	1869 (16.8)	5504 (16.3)	5501 (16.3)	1777 (16.2)	3646 (16.5)	3644 (16.5)
Ethnicity						
White	9225 (82.8)	28,185 (83.3)	28,166 (83.3)	9030 (82.3)	18,255 (82.6)	18,250 (82.6)
Other	1911 (17.2)	5640 (16.7)	5637 (16.7)	1946 (17.7)	3857 (17.4)	3857 (17.4)

Data reported as number (percentage) or mean ± SD

^a^IMD score in deciles

Complete-case data (i.e. non-missing) are shown—there was one missing information for inpatient death (age), 41 for readmission (one age and 40 IMD-10) and 68 for length of hospital stay (one age, 34 IMD-10, 27 time to discharge and six both IMD-10 and time to discharge)

**Table 2:** Model performance indices in derivation and validation samples

Model	Derivation			Validation			
	Nagelkerke *R* ^2^ (%)	C-index (95% CI)	Bias-corrected C-index	Nagelkerke *R* ^2^ (%)	C-index (95% CI)	Calibration slope	Calibration intercept
Inpatient death							
Base	12.1	0.77 (0.75, 0.80)	0.75	8.1	0.74 (0.71, 0.76)	0.77	0.00
Disease	11.8	0.78 (0.75, 0.80)	0.77	9.0	0.74 (0.72, 0.77)	0.86	-0.01
One month readmission							
Base	1.0	0.57 (0.56, 0.59)	0.56	0.5	0.55 (0.54, 0.57)	0.70	0.03
Disease	0.9	0.57 (0.56, 0.58)	0.56	0.4	0.55 (0.53, 0.56)	0.66	0.03
24 h discharge							
Base	10.6	0.68 (0.67, 0.69)	0.68	8.4	0.66 (0.65, 0.67)	0.83	0.04
Disease	11.1	0.69 (0.68, 0.69)	0.69	8.9	0.67 (0.66, 0.68)	0.82	0.05

### Model development and internal validation

The base and disease models for inpatient death were developed from 11,136 admissions and 296 (2.7%) deaths (Table 1). Age and Charlson score were significantly associated with the risk of inpatient death in both the base and disease models (Fig. 1 and ESM Table 2). Discrimination was very similar comparing the two models: the base model showed a C-index of 0.77 (95% CI 0.75, 0.80), with minimal over-fitting in bootstrap validation (bias-corrected C-index 0.75), while the disease model achieved a C-index of 0.78 (0.75, 0.80) with a bias-corrected value of 0.77 (Table 2). The prognostic models for one month readmission were derived from 1789 one month readmissions among 33,825 admissions (5.3%, Table 1). Ethnicity and region were significantly associated with risk of readmission in both the base and disease model. Discriminations were modest, being C-index 0.57 (0.56, 0.59) and 0.57 (0.56, 0.58) for the base and disease models, respectively; bias-corrected C-indices yielded similar results (Table 2). Finally, prognostic models for 24 h discharge were developed from 8396 24 h discharge among 33,803 admissions (24.8%). All variables of the base model were associated with 24 h discharge. In the disease model, 15 further variables were included; of which, 12 were associated with the outcome (ESM Table 2). C-indices were 0.68 (0.67, 0.69) for the base model and 0.69 (0.68, 0.69) for the disease model, with similar bias-corrected values (Table 2). Both models showed good calibration for inpatient death and 24 h discharge; conversely, one month readmission models had poor calibration, with no spread between deciles of predicted risk (Fig. 2).

### Temporal validation

The temporal validation of the two models showed values that were slightly lower than those obtained in the derivation sample and very similar when comparing base and disease models (Table 2). For inpatient death (296 events among 10,976 admissions, 2.7%; Table 1), C-indices were 0.74 (0.71, 0.76) for the base and 0.74 (0.72, 0.77) for the disease model (Table 2). Corresponding values for one month readmission (1207 events among 22,112 admissions, 5.5%; Table 1) were 0.55 (0.54, 0.57) and 0.55 (0.53, 0.56), and for 24 h discharge (5363 events among 22,107 admissions, 24.3%; Table 1), 0.66 (0.65, 0.67) and 0.67 (0.66, 0.68) (Table 2). Calibration plots showed good agreement between observed and predicted risk for inpatient death and 24 h discharge; however, a slightly higher predicted than observed risk was evident for the base model in the last (tenth) risk group (Fig. 2).

### Individual risk calculator

Coefficients obtained in the logistic regressions for inpatient death and 24 h discharge were used to develop an Excel calculator (see ESM) to estimate individual absolute predicted risk based on variables included in the base model (a mobile/desktop app is in production). For both outcomes, models were recalibrated using the calibration slope and intercept estimated in the validation samples (Table 2). We did not include one month readmission because of the poor performance of models for this outcome, and developed the calculator using only base models following criteria of parsimony and simplicity, and given the negligible differences in the performance between the base and disease models. The calculator allows the input of individual data on age, sex, ethnicity, Charlson score and England postcode (for social deprivation) for two patients to visually inspect the impact of changing a single variable. The calculator can be developed in other graphical interfaces by using data reported in Fig. 1 (coefficients), Table 2 (calibration slope and intercept) and ESM Excel file (spline transformation of age).

## Discussion

### Main findings

Using a large sample of hospital admissions for hypoglycaemia in England, we developed, internally and temporally validated and calibrated two prognostic models for length of hospital stay, inpatient death and readmission. The two models performed well in terms of fitting (*R* ^2^ similar to risk models in other clinical setting [23, 24]) and calibration, and did not meaningfully differ in the prediction of inpatient death and length of hospital stay, defined in this study as same-day discharge. Conversely, models failed to accurately predict one month readmission for hypoglycaemia. In fact, the same variables used for inpatient death and length of stay did not accurately predict the risk for one month readmissions, underlying the possibility that other, unmeasured factors are more relevant in identifying patients at higher risk of recurrent admissions for hypoglycaemia. For all outcomes, model performances were similar in temporal validations. These analyses allowed the development of a tool to assess individual risk based on basic information that are routinely collected in patients admitted to NHS hospital trusts in England.

### Interpretation in the context of available evidence

In the last decade, hospital admissions for hypoglycaemia have consistently increased in England, as well as in the USA [11, 15]. Although only a fraction of hypoglycaemic episodes result in hospitalisation, admissions are generally reserved for patients potentially at higher risk of complications and have considerable resource implications for national healthcare systems [4]. Studies have also shown that a significant proportion of admissions for hypoglycaemia occur in people previously admitted for the same reason [11, 15]. Therefore, the availability of prognostic models for length of hospital stay, risk of death and hospital readmission may be a useful tool to support clinicians and decision makers.

In recent years, various clinical risk models for inpatient death have been developed and validated in different clinical settings, including people with myocardial infarction [6], valve replacement [7] or abdominal aortic aneurism [8], or those admitted to intensive care units [25]. Similarly, validated models for readmissions are available for all-cause and cause-specific readmissions, such as cardiovascular, gastrointestinal or pulmonary diseases [9, 26–30]. More limited, on the other hand, are validated models for length of hospital stay for patients with, for example, chronic obstructive pulmonary disease [31], gastrointestinal bleeding [32] or stroke [10]. Studies aiming to develop clinical prediction models are appreciably different in terms of variable accessibility, model specification procedures, temporal and geographical settings and, most importantly, population studied. It is therefore not surprising that the final variables included in the models, the strength of their associations with outcomes and the occurrence of the outcomes are inconsistent across studies. This is in part due to differences in the aetiology and pathophysiology of diseases (which could influence, for example, the selection of variables) as well as to differences in their severity (for example, risk of inpatient death following decompensated heart failure vs hypoglycaemia). Therefore, the precise definition of a homogeneous population to whom the prediction models apply is of crucial importance.

### Strength and limitations

To our knowledge, no model has been developed to date to predict hospital outcomes in patients admitted for hypoglycaemia. A major strength of this study is the availability of a large nationwide database with detailed information on hospital admissions. Furthermore, information was missing only in a very low proportion of admissions, and models were internally and temporally validated.

At the same time, several points should be considered for the interpretation of these findings. First, in all models we used only variables available in the HES database. We may not have included important prognostic variables that could be particularly relevant for one month readmission. Insulin therapy and diabetes duration are related to a higher risk of severe hypoglycaemia and might confound the association between factors included in the analysis, such as age and comorbidities, and risk of readmission [33]. Lack of detailed data on glucose-lowering therapies could explain the low performance of models for this outcome. Similarly, lack of information on attendance at educational programmes to avoid recurrent severe hypoglycaemia might have influenced our results. It should be noted, however, that a recent systematic review has confirmed initial observations about the poor to moderate performance of risk models for one month hospital readmissions [28], even in those including an extensive panel of potential predictors [9]. Moreover, although several studies have evidenced multiple clinical risk factors for severe hypoglycaemia, the large majority of these analyses reported only associations that do not necessarily translate into better prognostic ability [34].

Second, the ICD-10 codes E10+ (insulin-dependent diabetes mellitus) and E11+ (non-insulin-dependent diabetes mellitus) have been used only to define the population under investigation (people with diabetes) and could not be considered as a proxy of treatment. Indeed, it is possible that individuals with insulin-treated type 2 diabetes have been coded as E11+. Conversely, while in principle E10+ and E11+ should respectively identify individuals with type 1 and type 2 diabetes, we could not exclude that some E10+ patients had insulin-treated type 2 diabetes. Finally, for some patients the HES data has inconsistent coding of E10+/E11+ over time (change of diabetes type). Therefore, we could not clearly separate the two groups for the analyses as they included non-well-phenotyped patients.

Third, variable selection in prognostic models is well recognised as the most difficult step in model development. At two extremes, selection of variables can be based only on the expert knowledge of subject matter or only on statistical methods, although the latter approach has been criticised for unstable selection of predictors and bias estimation of associations [20]. In this analysis, we developed a simple model, responding to criteria of parsimony and clinical knowledge, based on six simple items of information, and a second model, with more detailed specification of comorbidities, based on a statistical method to define the final set of variables. The performance of the two models, however, was very similar and justified the use of the variables in the base model for predicting individual risk.

Fourth, HES data are routinely collected for administrative rather than research purposes; as such, there is some potential for inaccuracies in data collection and recording.

Finally, we only considered death occurring during hospitalisation and not short- or long-term mortality after discharge, and investigated readmissions only for hypoglycaemia.

### Clinical and research implications

These prediction models were developed to estimate individual-level risk using simple clinical and demographic data. The models for length of stay and inpatient death performed well and, along with clinical judgement, could be used by decision makers to personalise targets and strategies. On the other hand, models failed to predict hospital readmission accurately. Given the substantial cost associated with hospital readmissions in the UK and the high prevalence of one month readmission in patients admitted for hypoglycaemia [11, 15, 35], further studies are warranted to address this important knowledge, clinical and public health gap.

Notwithstanding the importance and the implications of length of stay as a quality indicator across hospitals [36], there are still methodological uncertainties about the best modelling approach to analyse such data and further research is required. Logistic regression estimating discharge at meaningful time points, time-to-event analysis or mixture models have been suggested, with unclear advantages in simulations studies of one method over another [37–39]. Accounting for HES database characteristics, we opted to perform a logistic regression using 24 h as the specific time point. Indeed, in HES length of stay can be calculated as the difference between two dates, thus resulting in admissions of length of zero (24 h discharge, i.e. same date for entry and exit) or multiples of 1 day. As about 25% of discharges occurred within 24 h, time-to-event analysis was not a suitable approach to analyse these data. In similar circumstances where a significant proportion of discharges occur within 24 h, a more detailed description of length of hospitalisation with time-to-event analysis is possible only if length of stay is reported in fraction of day (i.e. hours).

We performed a validation of the models for admissions during two different periods (temporal validation). These results, therefore, pertain only to admissions for hypoglycaemia in England and full external validation (temporal and spatial) is required to validate models accounting for geographical and temporal differences.

### Conclusion

A prediction model for risk of inpatient death and 24 h discharge in individuals admitted to hospital for hypoglycaemia has been developed and validated. Individual risk can be estimated using simple information that is collected routinely in hospitalised patients. While further studies are required to validate this model and to assess the relevance of glucose-lowering therapies in risk prediction, this simple and pragmatic tool can improve the quality of care through personalised approaches and optimise resource allocation.

**Note added in proof**

During the review process of the manuscript, this research has been accepted as:

1. Poster at the Diabetes UK Professional Conference (8–10 March 2017, Manchester, UK)

2. Oral presentation at the Informatics for Health Congress (24–26 April 2017, Manchester, UK)

## Electronic supplementary material

ESM 1 (PDF 7.19 mb)

ESM 2 (XLSX 7371 kb)

## Abbreviations

HES: Hospital Episode Statistics
IMD: Index of multiple deprivation
NHS: National Health Service
