# Supplementary material for: Predicting hospital stay, mortality and readmission in people admitted for hypoglycaemia: prognostic models derivation and validation
Source: Diabetologia. 2017 Mar 17;60(6):1007–15. doi: 10.1007/s00125-017-4235-1 (PMC5423930; doi:10.1007/s00125-017-4235-1)
Supplement: Supplementary file 1 — (PDF 7.19 mb) [file 125_2017_4235_ESM1_ESM.pdf]

Electronic Supplementary Material

Predicting hospital stay, mortality, and readmission in people admitted for hypoglycaemia

Prognostic models derivation and validation

Francesco Zaccardi[1], David R Webb[1], Melanie J Davies[1], Nafeesa N Dhalwani[1], Laura J Gray[2], Sudesna Chatterjee[1], Gemma Housley[3], Dominick Shaw[3 4], James W Hatton[3], Kamlesh Khunti[1]

1 Diabetes Research Centre, University of Leicester, Leicester, England

2 Department of Health Sciences, University of Leicester, Leicester, England

3 Nottingham University Hospitals & East Midlands Academic Health Science Network, Nottingham, England

4 Nottingham Respiratory Research Unit, University of Nottingham, Nottingham, England

ESM Table 1: Outcome-specific top 20 most common diseases (ICD code position 2nd to 6th)

Inpatient mortality					Included*
**ICD-10**	**Disease**	**N**	**%**	**Cumulative %**	
E119	Non-insulin-dependent diabetes mellitus without complications	13,455	14.3	14.3	✓
I10X	Essential (primary) hypertension	6,852	7.3	21.5	✓
E109	Insulin-dependent diabetes mellitus without complications	5,892	6.2	27.7	
I48X	Atrial fibrillation and flutter	2,138	2.3	30.0	
Y423	Insulin and oral hypoglycaemic [antidiabetic] drugs	1,938	2.1	32.1	
N179	Acute renal failure, unspecified	1,748	1.9	33.9	✓
N390	Urinary tract infection, site not specified	1,741	1.8	35.8	
N189	Chronic renal failure, unspecified	1,601	1.7	37.5	
I259	Chronic ischaemic heart disease, unspecified	1,573	1.7	39.1	✓
E780	Pure hypercholesterolaemia	1,481	1.6	40.7	
I252	Old myocardial infarction	1,262	1.3	42.0	✓
E039	Hypothyroidism, unspecified	1,062	1.1	43.1	
F171	Mental & behavioural disease due use tobacco: harmful use	1,046	1.1	44.3	
J459	Asthma, unspecified	1,039	1.1	45.4	
H360	Diabetic retinopathy	1,025	1.1	46.4	
I500	Congestive heart failure	996	1.1	47.5	
J449	Chronic obstructive pulmonary disease, unspecified	992	1.1	48.5	
F03X	Unspecified dementia	970	1.0	49.6	
R296	Repeated falls	917	1.0	50.5	
I209	Angina pectoris, unspecified	900	1.0	51.5	
Other	-	45,814	48.5	100.0	
**TOTAL**		**94,442**	**100.0**		

One month readmission and 24hr discharge					Included*	
**ICD-10**	**Disease**	**N**	**%**	**Cumulative %**	**Readmission**	**24hr discharge**
E119	Non-insulin-dependent diabetes mellitus without complications	34,191	14.8	14.8	✓	✓
I10X	Essential (primary) hypertension	16,521	7.1	21.9		✓
E109	Insulin-dependent diabetes mellitus without complications	15,034	6.5	28.4		✓
I48X	Atrial fibrillation and flutter	5,256	2.3	30.6		✓
Y423	Insulin and oral hypoglycaemic [antidiabetic] drugs	4,508	1.9	32.6		
N390	Urinary tract infection, site not specified	4,331	1.9	34.5		✓
E780	Pure hypercholesterolaemia	4,086	1.8	36.2		✓
N179	Acute renal failure, unspecified	3,460	1.5	37.7		✓
N189	Chronic renal failure, unspecified	3,105	1.3	39.1	✓	
I259	Chronic ischaemic heart disease, unspecified	3,100	1.3	40.4		✓
E039	Hypothyroidism, unspecified	2,857	1.2	41.6		✓
I209	Angina pectoris, unspecified	2,710	1.2	42.8		✓
I258	Other forms of chronic ischaemic heart disease	2,653	1.1	43.9		✓
J459	Asthma, unspecified	2,599	1.1	45.1		
F03X	Unspecified dementia	2,427	1.0	46.1		✓
I500	Congestive heart failure	2,365	1.0	47.1		✓
J449	Chronic obstructive pulmonary disease, unspecified	2,355	1.0	48.2		
H360	Diabetic retinopathy	2,319	1.0	49.2		✓
Z867	Personal history of diseases of the circulatory system	2,055	0.9	50.0		✓
F171	Mental & behavioural disease due use tobacco: harmful use	1,997	0.9	50.9		
Other	-	113,730	49.1	100.0		
**TOTAL**		**231,659**	**100.0**			

* Initial models included age (spline with 5 knots), sex, ethnicity, region, IMD-10, Charlson score, and outcome-specific top 20 most common diseases (admissions in 2013-2014 for inpatient mortality; 2010-2014 for readmission and 24hr discharge). Final models were defined with a stepwise back elimination and included variables indicated with ✓ (odds ratios reported in ESM Table 2).

ESM Table 2: Outcome-specific odds ratios of variables included in disease models

Variable		Odds Ratio (95% confidence interval)		
		Inpatient mortality	One month readmission	24hr discharge
**Age (unit increase)**		1.05 (1.04, 1.07)	0.99 (0.99, 1.00)	0.98 (0.98, 0.99)
**Sex (Male vs Female)**		-	-	1.15 (1.10, 1.22)
**Ethnicity (Other vs White)**		-	0.69 (0.59, 0.80)	1.12 (1.04, 1.21)
**Region (ref East-England)**				
East-Midlands		-	0.87 (0.68, 1.10)	0.88 (0.77, 1.00)
London		-	1.41 (1.15, 1.72)	1.48 (1.32, 1.66)
North-East		-	1.02 (0.80, 1.31)	1.26 (1.10, 1.44)
North-West		-	1.18 (0.98, 1.43)	1.19 (1.06, 1.32)
South-East		-	0.84 (0.68, 1.05)	1.36 (1.21, 1.52)
South-West		-	0.81 (0.64, 1.04)	1.26 (1.11, 1.42)
West-Midlands		-	0.94 (0.75, 1.17)	1.12 (0.99, 1.25)
Yorkshire-Humber		-	1.15 (0.93, 1.42)	0.88 (0.78, 1.00)
**IMD-10 (ref Least deprived 10%)**				
Less deprived 10-20%		-	-	0.92 (0.79, 1.07)
Less deprived 20-30%		-	-	1.05 (0.91, 1.21)
Less deprived 30-40%		-	-	0.99 (0.86, 1.14)
Less deprived 40-50%		-	-	0.91 (0.80, 1.05)
More deprived 10-20%		-	-	0.80 (0.70, 0.91)
More deprived 20-30%		-	-	0.94 (0.82, 1.07)
More deprived 30-40%		-	-	0.83 (0.72, 0.95)
More deprived 40-50%		-	-	0.94 (0.82, 1.08)
Most deprived 10%		-	-	0.87 (0.76, 0.99)
**Charlson score (unit increase)**		1.36 (1.29, 1.43)	1.03 (0.99, 1.06)	0.76 (0.74, 0.78)
**Presence of disease**	**ICD-10**			
Hypothyroidism, unspecified	E039	-	-	0.73 (0.56, 0.96)
IDDM without complications	E109	-	-	2.55 (1.24, 5.28)
NIDDM without complications	E119	2.43 (1.30, 4.54)	0.51 (0.26, 0.99)	0.47 (0.33, 0.66)
Pure hypercholesterolaemia	E780	-	-	0.82 (0.68, 1.00)
Unspecified dementia	F03X	-	-	0.61 (0.43, 0.87)
Diabetic retinopathy	H360	-	-	0.61 (0.36, 1.05)
Essential (primary) hypertension	I10X	0.68 (0.41, 1.14)	-	0.56 (0.48, 0.64)
Angina pectoris, unspecified	I209	-	-	0.71 (0.52, 0.96)
Old myocardial infarction	I252	0.41 (0.13, 1.33)	-	-
Other forms of chronic ischaemic heart disease	I258	-	-	0.56 (0.43, 0.74)
Chronic ischaemic heart disease, unspecified	I259	0.49 (0.18, 1.34)	-	0.54 (0.39, 0.76)
Atrial fibrillation and flutter	I48X	-	-	0.50 (0.39, 0.66)
Congestive heart failure	I500	-	-	0.66 (0.44, 0.98)
Acute renal failure, unspecified	N179	2.97 (1.14, 7.73)	-	0.20 (0.09, 0.46)
Chronic renal failure, unspecified	N189	-	1.51 (0.97, 2.34)	-
Urinary tract infection, site not specified	N390	-	-	0.24 (0.11, 0.52)
Personal history of diseases of the circulatory system	Z867	-	-	0.82 (0.67, 1.02)
**Constant (exp)**		0.00022	0.07933	1.42862

NIDDM: non-insulin-dependent diabetes mellitus; **IDDM:** insulin-dependent diabetes mellitus
